# FctClus: A Fast Clustering Algorithm for Heterogeneous Information Networks

**DOI:** 10.1371/journal.pone.0130086

**Published:** 2015-06-19

**Authors:** Jing Yang, Limin Chen, Jianpei Zhang

**Affiliations:** 1 Institute of Computer Science and Technology, Harbin Engineering University, Harbin, China; 2 Institute of Computer Science and Technology, Mudanjiang Teachers College, Mudanjiang, China; National Institute of Genomic Medicine, MEXICO

## Abstract

It is important to cluster heterogeneous information networks. A fast clustering algorithm based on an approximate commute time embedding for heterogeneous information networks with a star network schema is proposed in this paper by utilizing the sparsity of heterogeneous information networks. First, a heterogeneous information network is transformed into multiple compatible bipartite graphs from the compatible point of view. Second, the approximate commute time embedding of each bipartite graph is computed using random mapping and a linear time solver. All of the indicator subsets in each embedding simultaneously determine the target dataset. Finally, a general model is formulated by these indicator subsets, and a fast algorithm is derived by simultaneously clustering all of the indicator subsets using the sum of the weighted distances for all indicators for an identical target object. The proposed fast algorithm, FctClus, is shown to be efficient and generalizable and exhibits high clustering accuracy and fast computation speed based on a theoretic analysis and experimental verification.

## Introduction

Information networks are ubiquitous and include social information networks and DBLP bibliographic networks. Numerous studies on homogeneous information networks, which consist of a single type of data object, have been performed; however, little research has been performed on the clustering of heterogeneous information networks, which consist of multiple types of data objects. Clustering on a heterogeneous network may lead to better understanding the hidden structures and deeper meanings of the networks[1].

The star network schema is popular and important in the field of heterogeneous information networks. The star network schema includes one data object target type and multiple data object attribute types, whereby each relation is the target data objects and all attribute data objects linking to it.

Algorithms based on compatible bipartite graphs can effectively consider multiple types of relational data. Various classical clustering algorithms, such as algorithms based on semi-definite programming[2,3], algorithms based on information theory[4] and spectral clustering algorithms for multi-type relational data[5], have been proposed for heterogeneous data from the compatible point of view. These algorithms are generalizable, but the computational complexity of these algorithms is too great for use in clustering heterogeneous information networks.

Sun et al. presents an algorithm, NetClus[6], and a PathSim-based clustering algorithm[7] for clustering heterogeneous information networks. NetClus is effective for DBLP bibliographic networks, but the algorithm is not a general model for clustering other heterogeneous information networks; NetClus is not sufficiently stable. The concept behind NetClus is also used for clustering service webs[8,9]. The PathSim-based clustering algorithm requires a user guide, and the clustering quality reflects the requirements of users rather than the requirements of the network. ComClus[10] is a derivation algorithm of NetClus for use with hybrid networks that simultaneously include heterogeneous and homogeneous relations. NetClus and ComClus are not general and depend on the given application.

Dynamic link inference in heterogeneous networks[11] requires more accurate initial clustering. A high clustering quality is necessary for network analysis, but low computation speed is intolerable because of the large network scales involved. The accuracy of the LDCC algorithm[12] is improved, while both the heterogeneous and homogeneous data relations are explored. The CESC algorithm[13] is very effective for clustering homogeneous data using an approximate commute time embedding. A heterogeneous information network with a star network schema can transform into multiple compatible bipartite graphs from the compatible point of view. When the relation between any two nodes of the bipartite graph is presented with the commute time, the relation of both heterogeneous and homogenous data objects can be explored; the clustering accuracy can also be improved. The heterogeneous information networks are large but very sparse; therefore, the approximate commute time embedding of each bipartite graph can be quickly computed using random mapping and a linear time solver[14]. All of the indicator subsets in each embedding indicate the target dataset, and subsequently, a general model for clustering heterogeneous information networks is formulated based on all indicator subsets. All weighted distances between the indicators and the cluster centers in the respective indicator subsets are computed. All indicator subsets can be simultaneously clustered according to the sum of the weighted distances for all indicators for an identical target object. Based on the above discussion, an effective clustering algorithm, FctClus, which is based on the approximate commute time embedding for heterogeneous information networks, is proposed in this paper. The computation speed and clustering accuracy of FctClus are high.

## Methods

### Commute Time Embedding of the Bipartite Graph

Given two types of datasets, $X_{0}=\{x_{1}^{\left(0\right)},x_{2}^{\left(0\right)},\cdots ,x_{n_{0}}^{\left(0\right)}\}$ and $X_{1}=\{x_{1}^{\left(1\right)},x_{2}^{\left(1\right)},\cdots ,x_{n_{1}}^{\left(1\right)}\}$, the graph *G*_*b*_ = 〈*V*, *E*〉 is called a bipartite graph if *V*(*G*_*b*_) = *X*_0_ ∪ *X*_1_ and $E(G_{b})=\{\langle x_{i}^{\left(0\right)},x_{j}^{\left(1\right)}\}$, where 1 ≤ *i* ≤ *n*_0_, 1 ≤ *j* ≤ *n*_1_. $W_{n_{0}\times n_{1}}$ is the relation matrix between *X*_0_ and *X*_1_, where the element *w*_*ij*_ is the edge weight between $x_{i}^{\left(0\right)}$ and $x_{j}^{\left(1\right)}$. Then, the adjacency matrix of the bipartite graph *G*_*b*_ can be denoted as

$${\tilde{W}}_{n\times n}=\left[\begin{array}{cc} 0 & W_{n_{0}\times n_{1}}\\ {\left(W^{T}\right)}_{n_{1}\times n_{0}} & 0 \end{array}\right]$$

*D*_1_ and *D*_2_ are the diagonal matrices, where the diagonal element of *D*_1_ is $d_{i}=\sum_{j=1}^{n_{1}}w_{ij}$ and the diagonal element of *D*_2_ is $d_{j}=\sum_{i=1}^{n_{0}}w_{ij}$.

$$D=\left[\begin{array}{cc} D_{1} & 0\\ 0 & D_{2} \end{array}\right]$$

thus the Laplacian matrix of the bipartite graph *G*_*b*_ is $L=D-\tilde{W}$. *L* can be eigen-decomposed into *L* = ΦΛΦ^*T*^, where Λ = *diag*(*λ*_1_, *λ*_2_,⋯, *λ*_*n*_) is a diagonal matrix composed of the eigenvalues of *L* and *λ*_1_ ≤ *λ*_2_ ≤ ⋯ ≤ *λ*_*n*_, Φ = (*ϕ*_1_, *ϕ*_2_, ⋯, *ϕ*_*n*_) is an eigenmatrix and *ϕ*_*i*_ is an eigenvector corresponding to the eigenvalue *λ*_*i*_. Let *L* ^+^ be a pseudo-inverse matrix of *L* and $L^{+}=\sum_{i=2}^{n}\frac{1}{\lambda_{i}}\phi_{i}\phi_{i}^{T}$. The bipartite graph is also an undirected weighted graph. According to the literature[15], the commute time *c*_*ij*_ between nodes *i* and *j* of *G*_*b*_ can be computed by the pseudo-inverse matrix *L* ^+^.

$$c_{ij}=g_{v}(l_{ii}^{+}+l_{jj}^{+}-2l_{ij}^{+})=g_{v}{\left(e_{i}-e_{j}\right)}^{T}L^{+}(e_{i}-e_{j})$$ (1)

where $l_{ij}^{+}$ is the (*i*, *j*) element of *L* ^+^, *g*_*v*_ = ∑ *w*_*ij*_, *e*_*i*_ is a unit column vector in which the *i*-th element is 1; that is, $e_{i}={\left[\underset{1}{0},\cdots ,\underset{i-1}{0},\underset{i}{1},\underset{i+1}{0},\cdots ,\underset{n}{0}\right]}^{T}$.

According to the literature[15,16], the commute time *c*_*ij*_ between nodes *i* and *j* of *G*_*b*_ is

$$\begin{array}{l} c_{ij}=g_{v}{\left(e_{i}-e_{j}\right)}^{T}\;L^{+}(e_{i}-e_{j})\\ \;=g_{v}{\left(e_{i}-e_{j}\right)}^{T}\;\Phi \Lambda^{-1}\Phi^{T}(e_{i}-e_{j})\\ \;={\left[\sqrt{g_{v}}\Lambda^{-1/2}\Phi^{T}(e_{i}-e_{j})\right]}^{T}[\sqrt{g_{v}}\Lambda^{-1/2}\Phi^{T}(e_{i}-e_{j})] \end{array}$$

Thus, the commute time *c*_*ij*_ is the square pairwise Euclidean distance between the row vectors in the space ${\left(\sqrt{g_{v}}\Lambda^{-1/2}\Phi^{T}\right)}^{T}$ or the column vectors in the space $\sqrt{g_{v}}\Lambda^{-1/2}\Phi^{T}$ [13], ${\left(\sqrt{g_{v}}\Lambda^{-1/2}\Phi^{T}\right)}^{T}$or $\sqrt{g_{v}}\Lambda^{-1/2}\Phi^{T}$ is called the commute time embedding of the bipartite graph *G*_*b*_. *c*_*ij*_ is the average path length between two nodes rather than the shortest path between two nodes. Using the commute time for clustering the noisy data increases robustness and captures the complex clusters. Therefore clustering in the commute time embedding can also effectively capture the complex clusters. $\sqrt{g_{v}}\Lambda^{-1/2}\Phi^{T}$ is used in this paper. If a normal Laplacian matrix *L*_*n*_ = *D* ^−1/2^ *LD* ^−1/2^ is used, the commute time embedding is $\sqrt{g_{v}}\Lambda^{-1/2}\Phi^{T}D^{-1/2}$ [13].

### Approximate Commute Time Embedding of the Bipartite Graph

If directly computing $\sqrt{g_{v}}\Lambda^{-1/2}\Phi^{T}$ or $\sqrt{g_{v}}\Lambda^{-1/2}\Phi^{T}D^{-1/2}$, the process requires *O*(*n* ^3^) time for the eigen-decomposition of the Laplacian matrix *L* or *L*_*n*_. *n = n*_0_ *+n*_1_ is the number of nodes and *s* is the number of edges in the bipartite graph *G*_*b*_. According to the literature[17], if the edges in *G*_*b*_ are oriented and

$$B(i,j)=\{\begin{array}{l} \;1\;i\;\text{is tail},\;j\;\text{is head}\\ \;-1\;i\;\text{is head},\;j\;\text{is tail}\\ \;0\;\text{others} \end{array}$$

where *i* and *j* are nodes of *G*_*b*_, then *B*_*s*×*n*_ is a directed edge-node incidence matrix. Using ${\hat{W}}_{s\times s}$ as a diagonal matrix whose entries are the edge weights, thus $L=B^{T}\hat{W}B$. Furthermore,

$$\psi =\sqrt{g_{v}}{\hat{W}}^{1/2}BL^{+}\in R^{s\times n}$$ (2)

thus, *ψ* is the commute time embedding of the bipartite graph *G*_*b*_, where the square root of the commute time is the Euclidean distance between *i* and *j* in *ψ* because

$$\begin{array}{l} c_{ij}=g_{v}{\left(e_{i}-e_{j}\right)}^{T}L^{+}(e_{i}-e_{j})\\ \;=g_{v}{\left(e_{i}-e_{j}\right)}^{T}L^{+}LL^{+}(e_{i}-e_{j})\\ \;=g_{v}{\left(e_{i}-e_{j}\right)}^{T}L^{+}B^{T}\hat{W}BL^{+}(e_{i}-e_{j})\\ \;={\left[\sqrt{g_{v}}{\hat{W}}^{1/2}BL^{+}(e_{i}-e_{j})\right]}^{T}[\sqrt{g_{v}}{\hat{W}}^{1/2}BL^{+}(e_{i}-e_{j})] \end{array}$$

According to the literature[18], given vectors *v*_1_,⋯, *v*_*n*_ ∈ *R* ^*s*^ and *ε* > 0, $Q_{k_{r}\times s}$ is a random matrix of row vectors, where $Q(i,j)=\pm 1/\sqrt{k_{r}}$ is equivalent when *k*_*r*_ = *O*(log *n* / *ε* ^2^). With probability 1−1 / *n*, at least

$$(1-\varepsilon )||\;v_{i}-v_{j}\;|{|}^{2}\leq ||\;Qv_{i}-Qv_{j}\;|{|}^{2}\leq (1+\varepsilon )||\;v_{i}-v_{j}\;|{|}^{2}$$ (3)

for all pairs.

Therefore, given the bipartite graph *G*_*b*_ with *n* nodes and *s* edges, *ε* > 0, and a matrix $Y_{k_{r}\times n}=\sqrt{g_{v}}Q{\hat{W}}^{1/2}BL^{+}$ with probability of at least 1−1 / *n*:

$$(1-\varepsilon )c_{ij}\leq ||\;Y(e_{i}-e_{j})\;|{|}^{2}\leq (1+\varepsilon )c_{ij}$$ (4)

for any nodes *i*, *j* ∈ *G*_*b*_, where *k*_*r*_ = *O*(log *n* / *ε* ^2^).

The proof of Eq (4) comes directly from Eq (2) and Eq (3). *c*_*ij*_ ≈||*Y*(*e*_*i*_ − *e*_*j*_)||^2^ with an error *ε* based on Eq (4). If directly computing $Y_{k_{r}\times n}=\sqrt{g_{v}}Q{\hat{W}}^{1/2}BL^{+}$, *L* ^+^ must first be computed, but the computational complexity of directly computing *L* ^+^ is excessive. However, using the method in the literature[19,20] to compute $Y_{k_{r}\times n}$, the complexity is decreased. Let $\theta =\sqrt{g_{v}}Q({\hat{W}}^{1/2}B)$; then, *Y* = *θL* ^+^, which is equal to *YL* = *θ*. First, $\theta =\sqrt{g_{v}}Q({\hat{W}}^{1/2}B)$is computed, and then, *YL* = *θ*. Each row of *Y*, *y*_*i*_, is computed by solving the system *y*_*i*_ *L* = *θ*_*i*_, where *θ*_*i*_ is the *i-*th row of *θ*. The linear time solver of Spielman and Teng[19,20] requires only $\tilde{O}(s)$ time to solve the system. Because ${\left\|y_{i}-{\hat{y}}_{i}\right\|}_{L}\leq \varepsilon {\left\|y_{i}\right\|}_{L}$ [17], where ${\hat{y}}_{i}$ is the solution, *y*_*i*_ *L* = *θ*_*i*_ using the linear time solver. Then, [17]

$${\left(1-\varepsilon \right)}^{2}c_{ij}\leq ||\hat{Y}(e_{i}-e_{j})|{|}^{2}\leq {\left(1+\varepsilon \right)}^{2}c_{ij}$$

Therefore, $c_{ij}\approx ||\hat{Y}(e_{i}-e_{j})|{|}^{2}$ with an error bound of *ε* ^2^. The component of the algorithm for the approximate commute time embedding of the bipartite graph is illustrated as follows.

**Algorithm1 ApCte** (Approximate Commute Time Embedding of the Bipartite Graph)

1. input the relation matrix $W_{n_{0}\times n_{1}}$;
2. compute the matrices *B*, $\hat{W}$and *L* using $W_{n_{0}\times n_{1}}$;
3. compute $\theta =\sqrt{g_{v}}Q({\hat{W}}^{1/2}B)$;
4. compute each ${\hat{y}}_{i}$ using the system *y*_*i*_ *L* = *θ*_*i*_ by calling to the Spielman-Teng solver *k*_*r*_ times[14], 1 ≤ *i* ≤ *k*_*r*_;
5. output the approximate commute time embedding $\hat{Y}$.

All data objects of *X*_0_ and *X*_1_ are mapped into a common subspace $\hat{Y}$, where the first *n*_0_ column vectors of $\hat{Y}$ indicate *X*_0_ and the last *n*_1_ column vectors of $\hat{Y}$ indicate *X*_1_. The dataset is composed of the *n = n*_0_ *+n*_1_ column vectors of $\hat{Y}$ is called an indicator dataset. The input matrix $W_{n_{0}\times n_{1}}$ is a sparse matrix with *s* nonzero elements. Therefore, the complexity of computing the matrices *B*, $\hat{W}$and *L* in step 2 is *O*(2*s*) + *O*(*s*) + *O*(*n*). The sparse matrix *B* has 2*s* nonzero elements, and the diagonal matrix $\hat{W}$ has *s* nonzero elements. Computing $\theta =\sqrt{g_{v}}Q({\hat{W}}^{1/2}B)$ takes *O*(2*sk*_*r*_ + *s*) time in step 3. Because the linear time solver of Spielman and Teng[19,20] requires only $\tilde{O}(s)$ time to solve for each *y*_*i*_ of system *y*_*i*_ *L* = *θ*_*i*_, constructing $\hat{Y}$ takes $\tilde{O}(sk_{r})$ time in step 4. Therefore, the complexity of algorithm1, ApCte, is only *O*(2*s*) + *O*(*s*) +*O*(*n*) + *O*(2*sk*_*r*_ + *s*) + $\tilde{O}(sk_{r})$ = $\tilde{O}(4s+n+3sk_{r})$. In practice, *k*_*r*_ = *O*(log *n* / *ε* ^2^) is small and does not vary between different datasets. The indicator dataset includes low-dimensional homogeneous data; therefore, traditional algorithms can be used for the indicator dataset.

### A General Model Formulation

Given a dataset $\chi ={\left\{X_{t}\right\}}_{t=0}^{T}$ with *T*+1 types, where *X*_*t*_ is a dataset belonging to the *t-*th type, a weighted graph *G* = < *V*, *E*, *W* > on *χ* is called an information network; if *V*(*G*) = *χ*, the *E*(*G*) is a binary relation on *V* and *W*: *E* → *R* ^+^. Such an information network is called a heterogeneous information network when *T* ≥ 1 and a homogeneous information network when *T* = 0[6].

An information network *G* = < *V*, *E*, *W* > on *χ* is called a heterogeneous information network with a star network schema if ∀*e* = 〈*x*_*i*_, *x*_*j*_〉 ∈ *E*, *x*_*i*_ ∈ *X*_0_ and *x*_*j*_ ∈ *X*_*t*_ (*t* ≠ 0). *X*_0_ is the target dataset, and *X*_*t*_ (*t* ≠ 0) is the attribute dataset.

To derive a general model for clustering the target dataset, a heterogeneous information network with a star network schema using the dataset $\chi ={\left\{X_{t}\right\}}_{t=0}^{T}$ with *T*+1 types is given, where *X*_0_ is the target dataset and ${\left\{X_{t}\right\}}_{t=1}^{T}$ are the attribute datasets. $X_{t}=\{x_{1}^{\left(t\right)},x_{2}^{\left(t\right)},\cdots ,x_{n_{t}}^{\left(t\right)}\}$, where *n*_*t*_ is the object number of *X*_*t*_. $W^{\left(0t\right)}\in R^{n_{0}\times n_{t}}$denotes the relation matrix between the target dataset *X*_0_ and the attribute dataset *X*_*t*_, where the element $w_{ij}^{\left(0t\right)}$ denotes the relation between $x_{i}^{\left(0\right)}$ of *X*_0_ and $x_{j}^{\left(t\right)}$ of *X*_*t*_. If an edge between $x_{i}^{\left(0\right)}$ and $x_{j}^{\left(t\right)}$ exists, its edge weight is $w_{ij}^{\left(0t\right)}$. If no edge exists, $w_{ij}^{\left(0t\right)}$ = 0. *T* relation matrices ${\left\{W^{\left(0t\right)}\right\}}_{t=1}^{T}$ exist in the heterogeneous information network with a star network schema.

The target dataset *X*_0_ and the attribute dataset *X*_*t*_ constitute a bipartite graph, *G* ^(0*t*)^, which corresponds to the relation matrix *W* ^(0*t*)^. The indicator dataset $Y^{\left(0t\right)}=\{y_{1}^{\left(0t\right)},y_{2}^{\left(0t\right)},\cdots ,y_{n_{0}+n_{t}}^{\left(0t\right)}\}$ which also is the approximate commute time embedding of *G* ^(0*t*)^ can be quickly computed by ApCte, where the first *n*_0_ data of *Y* ^(0*t*)^ indicate *X*_0_ and the last *n*_*t*_ data of *Y* ^(0*t*)^ indicate the attribute dataset *X*_*t*_. $Y_{t}^{\left(0\right)}$consists of the first *n*_0_ data of *Y* ^(0*t*)^, and *Y* ^(*t*)^ consists of the last *n*_*t*_ data of *Y* ^(0*t*)^. $Y_{t}^{\left(0\right)}$and *Y* ^(*t*)^ are called the indicator subsets. $y_{i}^{\left(t\right)}\in Y_{t}^{\left(0\right)}$indicates the *i-*th object of *X*_0_ and is called an indicator for 1 ≤ *i* ≤ *n*_0_. There exists a one-to-one correspondence between the indicators of $Y_{t}^{\left(0\right)}$ and the objects of *X*_0_. Because *T* bipartite graphs correspond to *T* indicator datasets, the target dataset *X*_0_ is simultaneously indicated by the *T* indicator subsets ${\left\{Y_{t}^{\left(0\right)}\right\}}_{t=1}^{T}$, and each object of *X*_0_ is simultaneously indicated by *T* indicators.

*β* ^(*t*)^ is the weight of the relation matrix *W* ^(0*t*)^, where $\sum_{t=1}^{T}\beta^{\left(t\right)}=1$, *β* ^(*t*)^ > 0. The target dataset *X*_0_ is partitioned into *K* clusters. The indicators of ${\left\{Y_{t}^{\left(0\right)}\right\}}_{t=1}^{T}$, which indicate the identical object of *X*_0_, belong to *T* clusters. The *T* clusters are in *T* different indicator subsets and are denoted using the same label. Let

$$F=\sum_{t=1}^{T}\left(\beta^{\left(t\right)}\gamma \|Y_{t}^{\left(0\right)}-\omega^{\left(t\right)}\;{\|}^{2}\right)=\sum_{t=1}^{T}(\beta^{\left(t\right)}\sum_{i=1}^{n_{0}}(\gamma_{ij}\sum_{j=1}^{K}\|y_{i}^{\left(t\right)}-\omega_{j}^{\left(t\right)}\;{\|}^{2}))$$ (5)

where $\omega_{j}^{\left(t\right)}$ is the *j-*th cluster center of the indicator subset $Y_{t}^{\left(0\right)}$. There exists a one-to-one correspondence between the indicator function $\gamma ={\left\{\gamma_{ij}\right\}}_{i=1}^{n_{0}}$ and the objects of *X*_0_. If all indicators, ${\left\{y_{i}^{\left(t\right)}\right\}}_{t=1}^{T}$, that indicates the *i-*th object of *X*_0_ belong to the *j-*th cluster, *γ*_*ij*_ = 1; otherwise, *γ*_*ij*_ = 0.

If the objective function *F* in Eq (5) is minimized, the clusters of *X*_0_ are optimal from the compatible point of view because each indicator subset reflects the relation between the target dataset and the attribute dataset. Obviously, determining the global minimum of Eq (5) is NP hard.

### Derivation of Fast Algorithm for Clustering Heterogeneous Information Networks

The following steps allow for the local minimum of *F* in Eq (5) to be quickly achieved by simultaneously clustering all of the indicator subsets.

### Setting the Cluster Label

When given the cluster label of each indicator subset, the modeling process can be simplified. Suppose that the labels of the *K* clusters of each $Y_{t}^{\left(0\right)}$ are set. Let *q*_1_, *q*_2_ ∈ *X*_0_, $y_{1}^{\left(1\right)},y_{2}^{\left(1\right)}\in Y_{1}^{\left(0\right)}$, $\cdots y_{1}^{\left(T\right)},y_{2}^{\left(T\right)}\in Y_{T}^{\left(0\right)}$. ${\left\{y_{1}^{\left(t\right)}\right\}}_{\text{t}=1}^{T}$ indicate *q*_1_, and ${\left\{y_{2}^{\left(t\right)}\right\}}_{\text{t}=1}^{T}$ indicate *q*_2_. The clusters which indicators for an identical target object belong to have the same label. If one indicator of ${\left\{y_{1}^{\left(t\right)}\right\}}_{\text{t}=1}^{T}$ belongs to the *j-*th cluster, all of the other indicators of ${\left\{y_{1}^{\left(t\right)}\right\}}_{\text{t}=1}^{T}$ also belong to the *j-*th cluster in their respective indicator subset. If ${\left\{y_{1}^{\left(t\right)}\right\}}_{t=1}^{T}$ belongs to the *j-*th cluster, then all ${\left\{y_{2}^{\left(t\right)}\right\}}_{t=1}^{T}$ either belong to the *j-*th cluster in their respective indicator subset or none belong to the *j-*th cluster.

Each cluster of $Y_{t}^{\left(0\right)}$ has an initial center. *K* random objects are selected from the target dataset *X*_0_. The indicators indicating the *K* objects are taken as the initial cluster centers for each $Y_{t}^{\left(0\right)}$ and for the clusters whose center indicates an identical target object with the same label. Then, all of the other indicators for an identical target object only belong to the *j-*th cluster in each $Y_{t}^{\left(0\right)}$ or no indicators belong to the *j-*th cluster, where 1 ≤ *j* ≤ *K*. Therefore, the *K* clusters of ${\left\{Y_{t}^{\left(0\right)}\right\}}_{t=1}^{T}$ are set labels.

### The sum of the Weighted Distances

An object of *X*_0_ is indicated by *T* indicators. All of the *T* distances between the indicator and the center in each $Y_{t}^{\left(0\right)}$ affect the object allocation. The target object allocation is determined by the sum of the weighted distances for the *T* indicators. Setting *q*_*i*_ ∈ *X*_0_, $y_{i}^{\left(1\right)}\in Y_{1}^{\left(0\right)}$, $\cdots y_{i}^{\left(T\right)}\in Y_{T}^{\left(0\right)}$, ${\left\{y_{i}^{\left(t\right)}\right\}}_{\text{t}=1}^{T}$ indicates *q*_*i*_. The weighted distance between $y_{i}^{\left(t\right)}$ and the *j-*th cluster center in $Y_{t}^{\left(0\right)}$ is $\beta^{\left(t\right)}\|y_{\text{i}}^{\left(t\right)}-\omega_{j}^{\left(t\right)}{\|}^{2}$. The sum of the weighted distances is $dis=\sum_{t=1}^{T}(\beta^{\left(t\right)}\|y_{\text{i}}^{\left(t\right)}-\omega_{j}^{\left(t\right)}{\|}^{2})$, which determines the cluster that the object *q*_*i*_ belongs.

$$j=\text{arg min}\sum_{t=1}^{T}(\beta^{\left(t\right)}\|y_{\text{i}}^{\left(t\right)}-\omega_{j}^{\left(t\right)}{\|}^{2})$$ (6)

where *j* is the cluster label.

### The Local Minimum of *F*

*F* in Eq (5) can also be expressed as

$$F=\sum_{t=1}^{T}(\beta^{\left(t\right)}\sum_{i=1}^{n_{0}}(\gamma_{ij}\sum_{j=1}^{K}\|y_{i}^{\left(t\right)}-\omega_{j}^{\left(t\right)}{\|}^{2}))=\sum_{i=1}^{n_{0}}(\gamma_{ij}\sum_{j=1}^{K}\sum_{t=1}^{T}(\beta^{\left(\text{t}\right)}\|y_{i}^{\left(t\right)}-\omega_{j}^{\left(t\right)}{\|}^{2}))$$ (7)

Obviously, Eq (7) is another representation of Eq (5).

Given the initial centers ${\left\{\omega_{j}^{\left(t\right)}\right\}}_{j=1}^{K}$ and the cluster labels in the *T* indicator subsets ${\left\{Y_{t}^{\left(0\right)}\right\}}_{t=1}^{T}$, ${\left\{Y_{t}^{\left(0\right)}\right\}}_{t=1}^{T}$ is first partitioned by computing Eq (6) and setting *F* = *F*_0_ in Eq (7). The cluster centers of ${\left\{Y_{t}^{\left(0\right)}\right\}}_{t=2}^{T}$ remain the same, and *γ*_*ij*_ is unchanged. The new center ${\left\{{\hat{\omega }}_{j}^{\left(1\right)}\right\}}_{j=1}^{K}$ of each cluster in $Y_{1}^{\left(0\right)}$ is computed. The new center is the mean of all data of each cluster. The new centers ${\left\{{\hat{\omega }}_{j}^{\left(1\right)}\right\}}_{j=1}^{K}$ of $Y_{1}^{\left(0\right)}$ replace the old centers, and subsequently, Eq (7) is used to set *F* = *F*_1_. Then,

$$F_{1}=\sum_{i=1}^{n_{0}}(\gamma_{ij}\sum_{j=1}^{K}\left(\beta^{\left(1\right)}\|y_{i}^{\left(1\right)}-{\hat{\omega }}_{j}^{\left(1\right)}{\|}^{2}+\sum_{t=2}^{T}(\beta^{\left(\text{t}\right)}\|y_{i}^{\left(t\right)}-\omega_{j}^{\left(t\right)}{\|}^{2})\right))\leq F_{0}$$ (8)

proving

$$F_{1}=\sum_{i=1}^{n_{0}}(\gamma_{ij}\sum_{j=1}^{K}\left(\beta^{\left(1\right)}\|y_{i}^{\left(1\right)}-{\hat{\omega }}_{j}^{\left(1\right)}{\|}^{2}+\sum_{t=2}^{T}(\beta^{\left(\text{t}\right)}\|y_{i}^{\left(t\right)}-\omega_{j}^{\left(t\right)}{\|}^{2})\right))$$

Because only the new centers ${\left\{{\hat{\omega }}_{j}^{\left(1\right)}\right\}}_{j=1}^{K}$ of $Y_{1}^{\left(0\right)}$ replace the old centers, *γ*_*ij*_ remains unchanged. Therefore

$$F_{1}=\sum_{i=1}^{n_{0}}(\gamma_{ij}\sum_{j=1}^{K}(\beta^{\left(1\right)}\|y_{i}^{\left(1\right)}-{\hat{\omega }}_{j}^{\left(1\right)}{\|}^{2}))+\sum_{i=1}^{n_{0}}(\gamma_{ij}\sum_{j=1}^{K}\sum_{t=2}^{T}(\beta^{\left(\text{t}\right)}\|y_{i}^{\left(t\right)}-\omega_{j}^{\left(t\right)}{\|}^{2}))$$

Because the cluster centers of ${\left\{Y_{t}^{\left(0\right)}\right\}}_{t=2}^{T}$ also remain unchanged, $\sum_{i=1}^{n_{0}}(\gamma_{ij}\sum_{j=1}^{K}\sum_{t=2}^{T}(\beta^{\left(\text{t}\right)}\|y_{i}^{\left(t\right)}-\omega_{j}^{\left(t\right)}{\|}^{2}))$ is constant, and $\sum_{i=1}^{n_{0}}(\gamma_{ij}\sum_{j=1}^{K}\left(\beta^{\left(1\right)}\|y_{i}^{\left(1\right)}-{\hat{\omega }}_{j}^{\left(1\right)}{\|}^{2})\right)$ $\leq \sum_{i=1}^{n_{0}}(\gamma_{ij}\sum_{j=1}^{K}\left(\beta^{\left(1\right)}\|y_{i}^{\left(1\right)}-\omega_{j}^{\left(1\right)}{\|}^{2})\right)$. Subsequently,

$$F_{1}\leq \sum_{i=1}^{n_{0}}(\gamma_{ij}\sum_{j=1}^{K}(\beta^{\left(1\right)}\|y_{i}^{\left(1\right)}-\omega_{j}^{\left(1\right)}{\|}^{2}))+\sum_{i=1}^{n_{0}}(\gamma_{ij}\sum_{j=1}^{K}\sum_{t=2}^{T}(\beta^{\left(\text{t}\right)}\|y_{i}^{\left(t\right)}-\omega_{j}^{\left(t\right)}{\|}^{2}))=F_{0}$$

Thus, the cluster centers of $Y_{1}^{\left(0\right)}$, for *F*_1_ ≤ *F*_0_, are replaced.

The new centers ${\left\{{\hat{\omega }}_{j}^{\left(1\right)}\right\}}_{j=1}^{K}$ of $Y_{1}^{\left(0\right)}$ replace the old centers, while the centers of ${\left\{Y_{t}^{\left(0\right)}\right\}}_{t=2}^{T}$ remain unchanged. Re-clustering ${\left\{Y_{t}^{\left(0\right)}\right\}}_{t=1}^{T}$ using Eq (6), where the corresponding value is *F* = *F*_2_ in Eq (7), gives *F*_2_ ≤ *F*_1_.

Partitioning ${\left\{Y_{t}^{\left(0\right)}\right\}}_{t=1}^{T}$ using Eq (6) computes the new cluster centers ${\left\{{\hat{\omega }}_{j}^{\left(1\right)}\right\}}_{j=1}^{K}$ of $Y_{1}^{\left(0\right)}$; the new centers replace the old centers ${\left\{\omega_{j}^{\left(1\right)}\right\}}_{j=1}^{K}$. Then, the same procedure is repeated for each ${\left\{Y_{t}^{\left(0\right)}\right\}}_{t=2}^{T}$. The value of *F* decreases in this case. The above procedures are repeated until *F* in Eq (7) converges; then, the local minimum of *F* in Eq (7) is obtained. The algorithm based on the approximate commute time embedding for heterogeneous information networks is shown below.

**Algorithm 2 FctClus** (Fast Clustering Algorithm based on the Approximate Commute Time Embedding for Heterogeneous Information Networks)

1. Input relation matrices ${\left\{W^{\left(0t\right)}\in R^{n_{0}\times n_{t}}\right\}}_{t=1}^{T}$, weights ${\left\{\beta^{\left(t\right)}>0\right\}}_{t=1}^{T}$ and cluster number *K*;
2. **for** *t* = 1 to *T* **do**
3. Compute indicator dataset *Y* ^(0*t*)^ of the bipartite graph corresponding to *W* ^(0*t*)^ using algorithm 1;
4. Constitute the indicator subset $Y_{t}^{\left(0\right)}$ that indicates *X*_0_;
5. end for
6. Initialize the *K* initial cluster centers ${\left\{\omega_{j}^{\left(t\right)}\right\}}_{j=1}^{K}$ of ${\left\{Y_{t}^{\left(0\right)}\right\}}_{t=1}^{T}$ and set the cluster label;
7. loop
8. **for** *t* = 1 to *T* **do**
9. Partition ${\left\{Y_{t}^{\left(0\right)}\right\}}_{t=1}^{T}$ into *K* clusters by computing Eq (6);
10. Re-compute the new cluster centers ${\left\{{\hat{\omega }}_{j}^{\left(t\right)}\right\}}_{j=1}^{K}$ of $Y_{t}^{\left(0\right)}$;
11. ${\left\{\omega_{j}^{\left(t\right)}={\hat{\omega }}_{j}^{\left(t\right)}\right\}}_{j=1}^{K}$;
12. end for
13. end loop
14. Output the clusters of *X*_0_.

The computational complexity of steps 2~5 is $\tilde{O}(\sum_{t=1}^{T}\left(4s_{t}+n_{t}+3s_{t}k_{r}\right))$ in algorithm 2, where *T* is the number of relational matrices in the heterogeneous information network and *k*_*r*_ is the data dimension of $Y_{t}^{\left(0\right)}$. *n*_*t*_ and *s*_*t*_ are the node number and edge number of the *t-*th bipartite graph, respectively. Step 6 requires only *O*(*K*) time; the time is constant. The object number of *X*_0_ is equal to the indicator number of each indicator subset, thus the computational complexity of steps 7~13 is *O*(*uTKk*_*r*_ *n*_0_), where *K* is the number of clusters of each $Y_{t}^{\left(0\right)}$; *n*_0_ is the data number of each $Y_{t}^{\left(0\right)}$; and *u* is the iteration number for *F* in Eq (7) convergence. Therefore, the computational complexity of algorithm 2, FctClus, is $\tilde{O}(\sum_{t=1}^{T}\left(4s_{t}+n_{t}+3s_{t}k_{r}\right))$ + *O*(*uTKk*_*r*_ *n*_0_), where *k*_*r*_ and *u* are small and *T* and *K* are constant.

## Experiments

### The Experimental Dataset

The experimental datasets are composed of real data selected from the DBLP data. The DBLP is a typical heterogeneous information network in computer science domain and contains 4 types of objects, including papers, authors, terms and venues. Two different-scaled heterogeous datasets called *S*_*small*_ and *S*_*large*_ respectively are used in experiments.

*S*_*small*_ is the small test dataset and is called the "four-area dataset", as in the literature[6]. *S*_*small*_ extracted from the DBLP dataset downloaded in 2011 contains four areas related to data mining: databases, data mining, information retrieval and machine learning. Five representative conferences for each area are chosen, and all papers and terms that appear in the titles are included. *S*_*small*_ is showed in S1 File.

*S*_*large*_ is the large test dataset and extracted from the Chinese DBLP dataset, which are sharing resources released by Institute of automation, Chinese Academy of Sciences. *S*_*large*_ includes 34 computer science journals, 16, 567 papers, 47, 701 authors and 52,262 terms(keywords). *S*_*large*_ is showed in S2 File.

When analyzing the papers, this object is the target dataset, and the other objects are the attribute datasets. There is no direct link between papers because the DBLP provided very limited citation information. When analyzing the authors, this object is the target dataset, while papers and venues are the attribute datasets. However, there is a direct link between authors because of the co-author relation between various authors; therefore, authors are another attribute dataset related to the target dataset.

The experiments are performed in the MATLAB 7.0 programming environment. The matlab source codes for our algorithm are showed in S3 File and are available online at https://github.com/lsy917/chenlimin, which include a main program and three function programs. FctClus.m is the main program which output the clusters of the object dataset, and ApCte.m, Prematrix.m and Net_Branches.m are function programs. The Koutis CMG solver[14] is used in all experiments as the nearly linear time solver to create the embedding. The solver uses symmetric, diagonally dominant matrices that are available online at http://www.cs.cmu.edu/~jkoutis/cmg.html.

### The Relational Matrix

Papers are the target dataset, while authors, venues and terms are the attribute datasets. *X*_0_ denotes papers, and *X*_1_, *X*_2_ and *X*_3_ denote authors, venues and terms, respectively. *W* ^(0*t*)^ is the relation matrix between *X*_0_ and *X*_*t*_, 1 ≤ *t* ≤ 3. The element of ${\left\{W^{\left(0t\right)}\right\}}_{t=1}^{3}$ is

$$w_{ij}^{\left(0t\right)}=\{\begin{array}{l} 1\;\text{if}\;i\in X_{0},j\in X_{1}\cup X_{2},\text{node}\;i\;\text{links to}\;j;\\ p\;\text{if}\;i\in X_{0},j\in X_{3},\;\text{node}\;i\;\text{appears}\;p\;\text{times in node}\;j;\\ \;0\;\text{otherwise}; \end{array}$$

When authors are the target dataset, papers and venues are the attribute datasets. Authors are also an attribute dataset because of the co-author relation existing between authors. *X*_0_ denotes authors when *X*_1_ and *X*_2_ denote papers and venues, respectively. *W* ^(0*t*)^ is the relation matrix between *X*_0_ and *X*_*t*_, 0 ≤ *t* ≤ 2. The element of ${\left\{W^{\left(0t\right)}\right\}}_{t=0}^{2}$ is

$$w_{ij}^{\left(0t\right)}=\{\begin{array}{l} 1\;\text{if}\;i\in X_{0},j\in X_{1}\cup X_{2},\text{node}\;i\;\text{links to}\;j;\\ p\;\text{if}\;i\in X_{0},j\in X_{0},\;\text{node}\;i\;\text{and}\;j\;\text{co - author}\;p\;\text{papers};\\ \;0\;\text{otherwise}; \end{array}$$

All the algorithms use the same relation matrix for all experiments.

### Parameter Analysis

#### Analysis of Parameter *k*_*r*_

The equation [13]

$$Accuracy=\frac{\sum_{i=1}^{n}\delta [map(c_{i})=label(i)]}{n}$$

is used to compute the clustering accuracy in the experiments, where *n* is the object number of dataset, *label(i)* is the cluster label, and *c*_*i*_ is the predicted label of an object *i*. *δ*(⋅) is an indicator function:

$$\delta (\cdot )=\{\begin{array}{l} 1\;map(i)=label(i)\\ 0\;map(i)\neq label(i) \end{array}.$$

*k*_*r*_ is small in practice, and minimal differences exist among the various datasets[13]. The literature[13] has proved that the accuracy curve is flat for clustering different homogeneous datasets when *k*_*r*_≥50.

Using the small dataset *S*_*small*_, the clustering accuracy as a function of *k*_*r*_ in a heterogeneous information network is studied.

**Fig 1:**
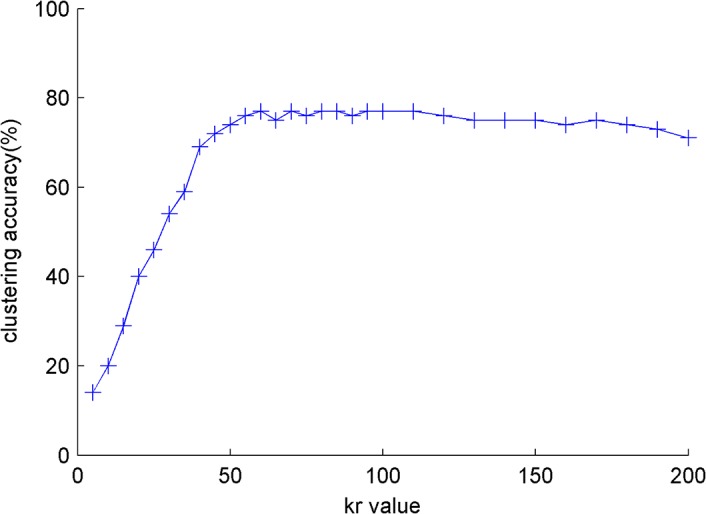
The influence of *k*_*r*_ for clustering papers on *s*_*small*._

**Fig 2:**
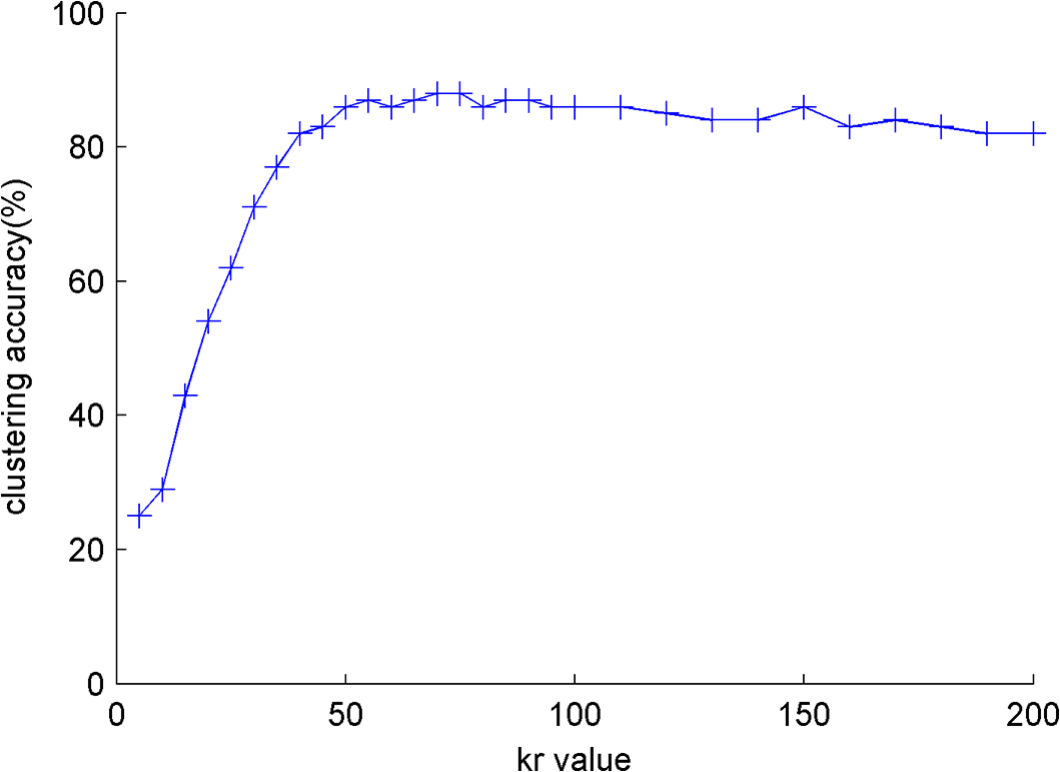
The influence of k_*r*_ for clustering authors on *s*_*small*._

An experiment with different *k*_*r*_ is conducted in the small dataset, *S*_*small*_. In the FctClus algorithm, the weight of ${\left\{W^{\left(0t\right)}\right\}}_{t=1}^{3}$ is taken as *β* ^(1)^ = 0.3, *β* ^(2)^ = 0.4 and *β* ^(3)^ = 0.3 for clustering papers; the weight of ${\left\{W^{\left(0t\right)}\right\}}_{t=0}^{2}$ is taken as *β* ^(1)^ = 0.4, *β* ^(2)^ = 0.2 and *β* ^(3)^ = 0.4 for clustering authors. The clustering accuracy is affected by *k*_*r*_, as shown in Fig 1 and Fig 2.

The parameter *k*_*r*_ is quite small because the accuracy curve is flat when *k*_*r*_ obtains a certain value. *k*_*r*_ = 60 is suitable for the dataset in the experiment. *k*_*r*_ is small and does not considerably affect the computation speed of FctClus. It is advantageous that FctClus is not sensitive to *k*_*r*_ in terms of both accuracy and performance. All weights of the relation matrix and *k*_*r*_ = 60 are studied in other experiments.

### Analysis of Iteration *u*

**Fig 3:**
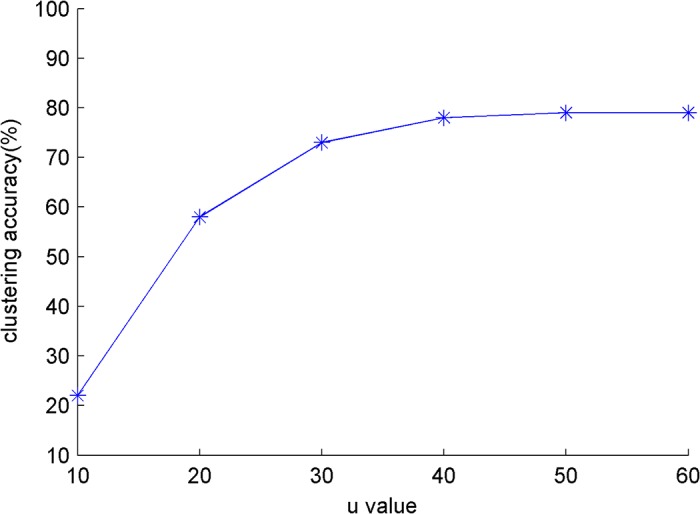
The influence of *u* for clustering papers on *s*_*small*._

**Fig 4:**
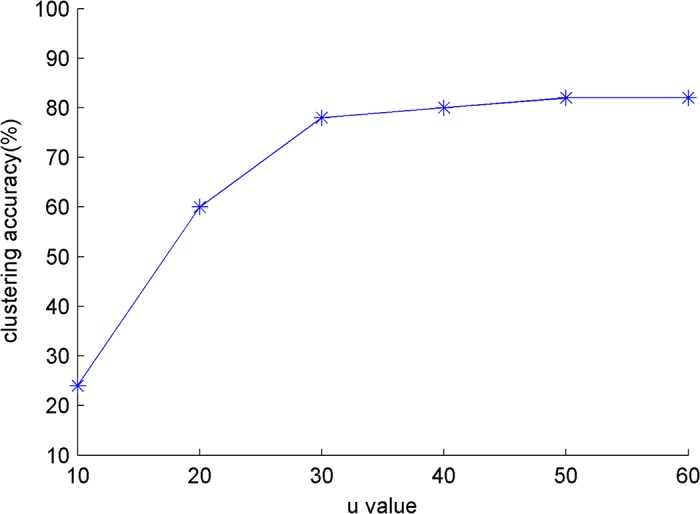
The influence of *u* for clustering authors on *s*_*small*._

An experiment is conducted in the small dataset *S*_*small*_ to compare the influence of iteration *u* on the clustering result, where *k*_*r*_ = 60. The influence of the iteration *u* on clustering papers and authors is shown in Fig 3 and Fig 4. The algorithm quickly convergences when *u* = 30. *u* = 40 is examined in the other experiments.

### Comparison of Clustering Accuracy and Computation Speed

The complexity of the algorithms is too high for large-scale networks based on semi-definite programming[2,3] and spectral clustering algorithms for multi-type relational data[5]. The low-complexity algorithms CIT[4], NetClus[6] and ComClus[10] are selected for comparison with the FctClus algorithm in terms of clustering accuracy and computation speed; the datasets *S*_*small*_ and *S*_*large*_ are also chosen for this experiment.

The initial cluster centers of FctClus or the initial cluster partitions of the other three algorithms are randomly selected 3 times. The best clustering accuracy of the 3 measurements is used as the clustering accuracy of the four algorithms, and the computation speed at this time is considered as the measured computation speed. The parameters in literature[6] are used as the parameters in NetClus, and the parameters in literature[10] are used as the parameters in ComClus in this experiment. The comparison results are shown in Table 1 and Table 2.

**Table 1:** Comparison of clustering accuracy (%).

target object &dataset	CIT	NetClus	ComClus	FctClus
Papers on *s*_*small*_	73.91	71.54	72.83	78.87
Authors on *s*_*small*_	74.41	69.13	74.91	81.33
Papers on *s*_*large*_	70.84	71.28	72.93	76.36
Authors on *s*_*large*_	71.02	68.29	73.01	77.94

**Table 2:** Comparison of computation speed(s).

target object &dataset	CIT	NetClus	ComClus	FctClus
Papers on *s*_*small*_	78.5	37.3	40.3	37.1
Authors on *s*_*small*_	79.8	36.9	39.8	38.3
Papers on *s*_*large*_	1469.3	802.6	827.3	808.4
Authors on *s*_*large*_	1484.7	743.7	781.4	774.9

The clustering accuracy of FctClus is the highest of all four algorithms. The clustering accuracy of CIT is lower than that of FctClus because the bipartite graphs of the heterogeneous information networks are sparse. The computational complexity of CIT is *O*(*n* ^2^), and the convergence speed of CIT is low when the heterogeneous information network is sparse. The clustering accuracy of NetClus is low because only heterogeneous relations are used. Homogeneous and heterogeneous relations are both used in ComClus; therefore, the accuracy of ComClus is higher than that of NetClus. FctClus is an algorithm based on commute time embedding. The data relations are explored using commute time and the direct relations of the target dataset are considered. FctClus is not affected by the sparsity of networks; thus, FctClus is highly accurate.

The computation speed of FctClus is nearly as fast as NetClus. The experiment demonstrates that FctClus is effective. FctClus is more universal and can be adapted for clustering any heterogeneous information network with a star network schema. However, NetClus and ComClus can only be adapted for clustering bibliographic networks because NetClus and ComClus depend on a ranking function of a specific application field.

### Comparison of Clustering Stability

To compare the stability of the FctClus, NetClus and CIT algorithms, the small dataset *S*_*small*_ is used for clustering papers in this experiment. ComClus is a derivation algorithm of NetClus; it has the same properties as NetClus. ComClus is not considered in this study.

**Fig 5:**
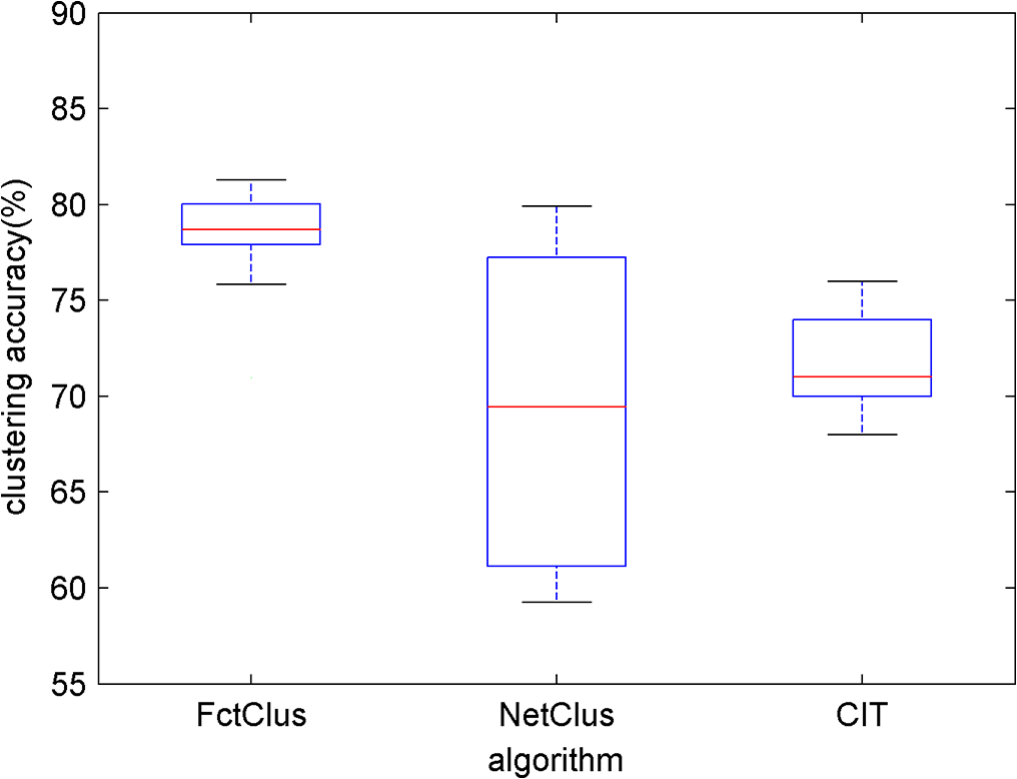
A stability comparison of the 3 algorithms for 10 times.

The initial cluster centers of FctClus and the initial cluster partitions of NetClus and CIT are randomly recorded 10 times, and the three algorithms are executed 10 times respectively. The clustering accuracy of the three algorithms for 10 times is shown in Fig 5. Although the computation speeds of FctClus and NetClus are both high, Fig 5 shows that the stability of FctClus is higher than that of NetClus and that the initial centers do not greatly impact the clustering result of FctClus. However, NetClus is very unstable, and the initial clusters greatly impact the clustering accuracy and convergence speed of NetClus. CIT is more stable than NetClus, but the clustering accuracy is low.

### Running Time Analysis of the FctClus Algorithm

The running time distributions of FctClus on the two datasets are shown in Table 3. The experimental data show that FctClus is effective. The running time for serial computing the three embedding is less than 50% of the total running time. When utilizing parallel computing for the three embedding, the computation speed is higher. When clustering indicator subsets in parallel, the computation speed may also be increased.

**Table 3:** Distribution of running time for FctClus.

target object &dataset	Embedding time(s)	Clustering time(s)	Total time(s)
Papers on *s*_*small*_	19.6	17.5	37.1
Authors on *s*_*small*_	18.1	20.2	38.3
Papers on *s*_*large*_	398.8	409.6	808.4
Authors on *s*_*large*_	382.4	392.5	774.9

## Conclusions

The relation between the original data described by the commute time guarantees the accuracy and performance of the FctClus algorithm. Because heterogeneous information networks are sparse, FctClus can use random mapping and a linear time solver[14] to compute the approximate commute time embedding, which guarantees the high computation speed. FctClus is effective and may be broadly implemented for large heterogeneous information networks, as demonstrated in theory and experimentally. The weight of the relation matrix impacts the target function, but the weight cannot be determined self-adaptively; this requires further research. The relations of data in the real world are typically high-order heterogeneous, so effective clustering algorithms for heterogeneous information networks with any schema will be studied in the future.

## Supporting Information

S1 File *S*_*small*_ dataset. (TXT)

S2 File *S*_*large*_ dataset. (TXT)

S3 File The matlab source codes for algorithm. (TXT)
